# Microsensor in Microbioreactors: Full Bioprocess Characterization in a Novel Capillary-Wave Microbioreactor

**DOI:** 10.3390/bios12070512

**Published:** 2022-07-11

**Authors:** Kevin Viebrock, Dominik Rabl, Sven Meinen, Paul Wunder, Jan-Angelus Meyer, Lasse Jannis Frey, Detlev Rasch, Andreas Dietzel, Torsten Mayr, Rainer Krull

**Affiliations:** 1 Institute of Biochemical Engineering, Technische Universität Braunschweig, 38106 Braunschweig, Germany; k.viebrock@tu-braunschweig.de (K.V.); p.wunder@tu-braunschweig.de (P.W.); jan-angelus.meyer@tu-braunschweig.de (J.-A.M.); l.frey@tu-braunschweig.de (L.J.F.); d.rasch@tu-braunschweig.de (D.R.); 2 Center of Pharmaceutical Engineering, Technische Universität Braunschweig, 38106 Braunschweig, Germany; s.meinen@tu-braunschweig.de (S.M.); a.dietzel@tu-braunschweig.de (A.D.); 3 Institute of Analytical Chemistry and Food Chemistry, Technische Universität Graz, 8010 Graz, Austria; dominik.rabl@tugraz.at (D.R.); torsten.mayr@tugraz.at (T.M.); 4 Institute of Microtechnology, Technische Universität Braunschweig, 38124 Braunschweig, Germany

**Keywords:** microbioreactor, optical sensor, capillary waves, glucose sensor, droplet cultivation

## Abstract

Microbioreactors (MBRs) with a volume below 1 mL are promising alternatives to established cultivation platforms such as shake flasks, lab-scale bioreactors and microtiter plates. Their main advantages are simple automatization and parallelization and the saving of expensive media components and test substances. These advantages are particularly pronounced in small-scale MBRs with a volume below 10 µL. However, most described small-scale MBRs are lacking in process information from integrated sensors due to limited space and sensor technology. Therefore, a novel capillary-wave microbioreactor (cwMBR) with a volume of only 7 µL has the potential to close this gap, as it combines a small volume with integrated sensors for biomass, pH, dissolved oxygen (DO) and glucose concentration. In the cwMBR, pH and DO are measured by established luminescent optical sensors on the bottom of the cwMBR. The novel glucose sensor is based on a modified oxygen sensor, which measures the oxygen uptake of glucose oxidase (GOx) in the presence of glucose up to a concentration of 15 mM. Furthermore, absorbance measurement allows biomass determination. The optical sensors enabled the characterization of an *Escherichia coli* batch cultivation over 8 h in the cwMBR as proof of concept for further bioprocesses. Hence, the cwMBR with integrated optical sensors has the potential for a wide range of microscale bioprocesses, including cell-based assays, screening applications and process development.

## 1. Introduction

Cultivation of microorganisms at the microscale is a promising alternative to conventional cultivation platforms such as shake flasks and lab-scale bioreactors in bioprocess development, high-throughput screenings, dose–response and other cell-based assays [1,2]. Therefore, research has focused on microscale cultivation platforms such as microbioreactors (MBRs) and droplet-based cultivation systems.

MBRs are cultivation platforms with a working volume <1 mL. A broad spectrum of MBRs with varying shapes, cultivation modes, mixing strategies, sensor integration and parallelization has been published recently. MBRs can be designed as microfluidic flow-through MBRs [1,3,4] and batch-mode MBRs such as microtiter plates [2,5,6,7], microbubble columns [8,9,10,11] or miniaturized stirred tank reactors [12,13]. The main advantage of MBRs compared to conventional cultivation platforms such as shake flasks is their comparably low volume combined with a high amount of online process data generated by integrated sensors. These characteristics allow simple automatization of a high number of parallel MBRs. Furthermore, cultivation at the microscale consumes less cultivation media and test substances, resulting in cost savings, especially for less available and expensive ingredients [14,15,16,17].

Another alternative to MBRs is a droplet-based cultivation system with even lower cultivation volumes. These systems can be divided into two main categories: sessile droplets and segmented-flow devices. Sessile droplets are placed on a flat surface, and each droplet acts as an individual reaction chamber. In contrast, segmented-flow devices consist of two immiscible fluids with droplets of cultivation media in an immiscible surrounding fluid. Various droplet-based cultivation systems have been reported recently [18]. One possibility for process characterization in these droplet-based cultivations is fluorescence measurement, e.g., of GFP-producing *Escherichia coli* strains [19].

Even though droplet-based cultivation systems contain a minimal volume of cultivation media and thereby allow high parallelization, biological process data provided by integrated sensors are still rare. However, process data are essential for all bioprocesses and therefore limit the application of droplet-based cultivation systems. In contrast, many MBRs have a high degree of sensor integration and thereby allow bioprocess characterization but contain a comparably high volume. This makes them less suitable for high parallelization and automatization and increases media and test substance consumption. Therefore, a 7 µL MBR was developed, which was previously presented by Frey et al. [20] and Meinen et al. [21] and is further optimized in this paper. This MBR is a promising tool combining minimal cultivation volume with integrated optical sensors for process characterization. The MBR consists of a photosensitive Foturan^®^ glass chip with an internal cavity holding a sessile droplet. The glass chip is fabricated by femtosecond laser direct writing, which is an ablation process forming a frustum-shaped cavity, as described by Meinen et al. [21]. By placing the MBR chip in a 3D-printed mounting, optical fibers for the sensor read-out can be guided to the specific position. Furthermore, evaporation of the cultivation droplet is minimized by internal water reservoirs in the mounting lid. The cultivation droplet is mixed by capillary waves induced by the vertical oscillation of the MBR platform, giving the reactor its name: the capillary-wave microbioreactor (cwMBR). At the resonance frequencies of the droplet, capillary waves with specific wave patterns are formed on droplet surfaces, leading to the mixing of the cwMBR volume, as described by Frey et al. [20]. Optimal mixing conditions with volumetric liquid-phase mass transfer coefficients (*k_L_a*) of more than 340 h^−^^1^ and mixing times of 2 s can be achieved at the resonance frequencies of the droplet. Furthermore, the mixing time and *k_L_a* can be tailored by adjusting different oscillation frequencies. To achieve ideal cultivation conditions, the cwMBR is placed in an incubation chamber with temperature and humidity control. Although dissolved oxygen (DO) and scattered light sensors for biomass determination were previously integrated in the cwMBR, process information about cultivation in the cwMBR is still scarce. Here, the integration of an optical pH and a novel glucose sensor in addition to the existing DO sensor is presented. Furthermore, the existing scattered light sensor was replaced here by a more robust and space-saving light absorbance sensor.

**Figure 1:**
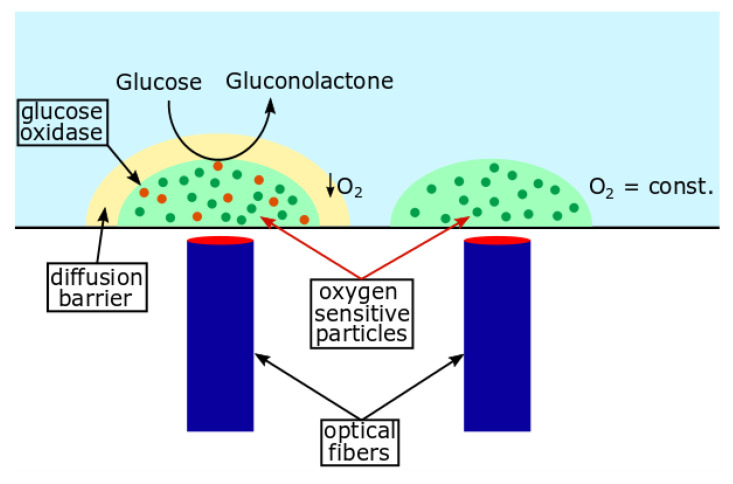
Working principle of the glucose sensor: glucose diffuses through the diffusion barrier and is oxidized aerobically by glucose oxidase to gluconolactone. Hence, the oxygen partial pressure in the glucose sensor declines, while it remains constant in a separated oxygen sensor. The difference in oxygen partial pressure between the two sensors is proportional to the glucose concentration. A read-out device is connected to the sensor spots via optical fibers.

Sensor integration in a microscale system is challenging because of the limited space in these systems. For example, the cwMBR bottom has a surface of only 4 mm^2^. Due to their minimal size, low-cost production and contactless read-out, optical sensors are well suited for application in MBRs. Potential optical sensors for application in microsystems have been reviewed by different authors [22,23,24,25]. As glucose is one of the most frequently used carbon sources in biotechnological processes, its quantification is of great importance at macro- and microscales. Besides optical sensors, electrochemical glucose sensors can be integrated into MBRs. Panjan et al. [26] developed an electrochemical glucose biosensor for MBR application, which consists of carbon electrodes covered with glucose oxidase as a biocatalyst and Prussian Blue as a mediator. Although the sensor was successfully applied in a 550 µL microbubble column bioreactor [8], it is too large for MBRs with a volume in the lower microliter scale, as presented in this work. Furthermore, this glucose sensor has a comparably low dynamic range up to 3 mM, which limits its applicability, as most cultivation media contain higher glucose concentrations. Therefore, optical glucose sensors are able to perform this task. Many optical glucose sensors have been developed for blood glucose measurement, e.g., in diabetes patients, as reviewed by Steiner et al. [27]. However, most of these sensors have a maximal measurable glucose concentration between 10 and 15 mM, as these concentrations are relevant for diabetes patients [28,29,30,31]. One example that appears to be transferable to MBR application was presented by Nacht et al. [32] with a maximal measurable glucose concentration of 20 mM. It combines an optical oxygen sensor with a separated glucose biosensor. Both sensors contain different phosphorescent porphyrin dyes for oxygen detection. Immobilized glucose oxidase (GOx) in the glucose sensors reduces oxygen to hydrogen peroxide depending on the glucose concentration in the sensor. Subsequently, the oxygen concentration in the glucose sensor declines proportionally to the glucose concentration, which can be measured by a read-out device. For application in the cwMBR, an optical glucose sensor with minimal size and maximal dynamic range and stability is necessary. Therefore, the glucose sensor reported by Nacht et al. [32] was modified for biotechnological processes and integrated into the cwMBR (Figure 1). Afterwards, it was fully characterized and applied in an *E. coli* culture as proof of concept for further application. The applicability of an optical glucose biosensor based on the oxygen uptake by GOx was previously demonstrated by Koštejnová et al. [33]. Nevertheless, an application at the microscale has not been reported so far. Furthermore, this sensor concept contains an optical oxygen sensor, which allows the measurement of DO, which is important for process characterization in the cwMBR as well.

For full process characterization, a pH sensor is necessary in addition to biomass, DO and glucose sensors. Due to the production or consumption of acid or alkaline by-products, pH shifts occur in most biotechnological cultivations. These pH shifts can have a great influence, e.g., on the growth rate or product formation [34,35,36]. Therefore, pH measurement is also essential in small- and large-scale bioreactors. An optical pH sensor based on near-infrared-emitting (NIR) aza-BODIPY dyes was applied for pH measurement in the cwMBR. Deprotonation of aza-BODIPYs leads to fluorescence quenching of the dye, which can be applied for pH measurement. This sensor combines a minimal size and contactless read-out with a high photo-stability, wide pH range depending on the number of applied aza-BODIPY dyes and sharp absorption and emission bands in the NIR region. NIR emission is beneficial due to the low background fluorescence of most biomolecules in this spectral region and less background scattering [37,38,39]. The sensor has previously been applied in several microfluidic devices [39,40,41,42] and bioprocesses [8,41,42,43], allowing precise pH measurement even in minimal volumes.

As the measurement of different process parameters in small-scale MBRs is still a challenge, the aim of this paper is the integration and application of four different optical sensors in a cwMBR. Besides established optical sensors for pH and oxygen, a novel glucose sensor is integrated. This glucose sensor is based on the oxygen uptake of GOx. As it has not been previously described, the glucose sensor is fully characterized in this paper. Furthermore, biomass is measured by absorption measurement, which replaces the previously applied and less stable scattered light measurement. As proof of concept for bioprocess characterization, all sensors were applied to describe the cultivation of *E. coli* in the cwMBR, which was compared to shake flask cultivation.

## 2. Materials and Methods

### 2.1. Manufacturing of the cwMBR and Surrounding Components

**Figure 2:**
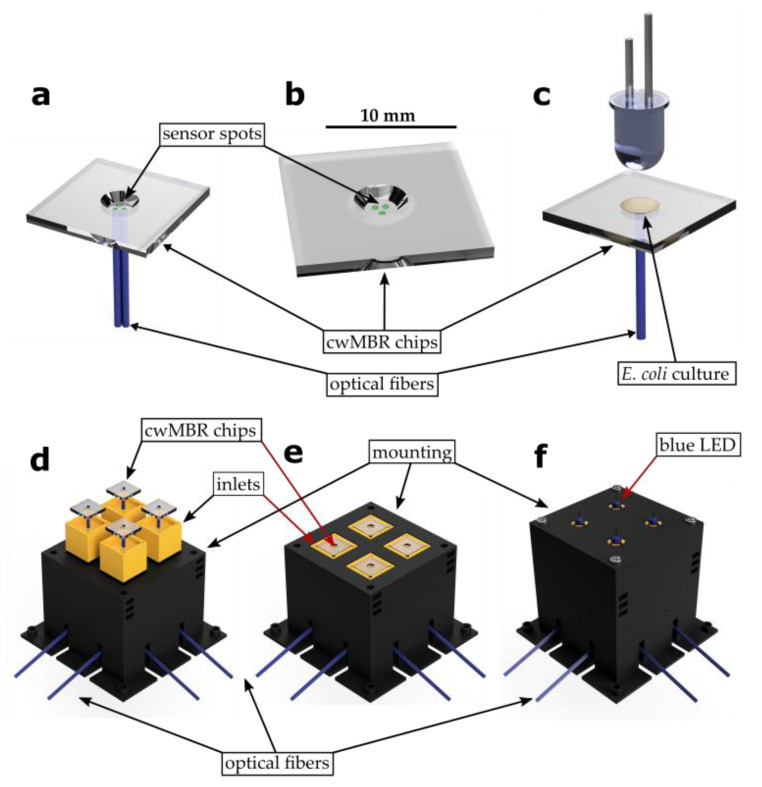
(**a**) Read-out of optical sensor spots in a cwMBR via optical fibers that are connected to a read-out device; (**b**) sensor spots on the bottom of the cwMBR; (**c**) absorption measurement in the cwMBR: a blue LED over the cwMBR is used as the light source. The unabsorbed light is measured by a spectrometer, which is connected to the cwMBR via optical fibers; (**d**) exploded view of the cwMBR mounting with cwMBRs in specific inlets for fixation of the cwMBR and optical fibers for sensor read-out; (**e**) assembled view of the cwMBR mounting; (**f**) cwMBR mounting with lid for minimal evaporation and integrated blue LEDs for absorbance measurement.

The cwMBR consists of a glass chip with a round, frustum-shaped cavity in its middle holding a 7 µL droplet of cultivation medium (Figure 2b). This glass chip was placed in a 3D-printed mounting, which ensures the fixation of the chip, sensor read-out via optical fibers and the minimization of evaporation (Figure 2e). For the mixing of the cultivation medium by capillary waves induced by vertical oscillation, as described by Frey et al. [20], the cwMBR mounting was screwed onto an exciter platform. The platform was placed in an incubation chamber to provide optimal cultivation conditions in the cwMBR.

The cwMBR was fabricated as previously described by Meinen et al. [21] by femtosecond laser direct writing in photosensitive Foturan^®^ glass (Schott, Mainz, Germany), forming a round cavity with an upper diameter of 4 mm and a depth of 1 mm. The optical transparent glass design enables the read-out of optical sensor spots from outside the cwMBR.

The cwMBR mounting was prepared by 3D printing (i3 Mega, Anycubic, Shenzhen, China) with black colored Polylactic acid (Getech, Shenzhen, China) to reduce light reflection by the mounting. For precise positioning of optical fibers for sensor read-out, the mounting contains more precisely 3D-printed inlets (Agilista 3200 W, Keyence, Osaka, Japan) made of AR-M2 polymer (Keyence, Osaka, Japan) (Figure 2d).

The mounting (Figure 2e) can hold up to four parallel cwMBRs, which are placed in the inlets of the mounting. The inlets contain channels for four optical fibers, which end directly under the cwMBR with its integrated sensor spots. The inlets were placed in the main body, which was covered by a lid to minimize evaporation (Figure 2f).

The lid forms a headspace with a minimal volume over the cultivation droplet. The headspace is humidified by moistened sponges to minimize evaporation. Due to their high surface area, evaporation from the sponges is favored over evaporation from the cultivation medium. Furthermore, the volume of the headspace over the cwMBR is minimized, so less liquid can evaporate. Moistened silicone mats were used as a sealant between the lid and main body, which were fixed by four screws. Gas exchange is facilitated by openings over the sponges. With the help of these measures, it was possible to reduce evaporation to <0.1 µL/h. The evaporation rate was determined by filling the cwMBR with 7 µL of water and measuring the remaining volume after 6 h under cultivation conditions (see Section 2.5) with a pipette (Reference 2, Eppendorf, Hamburg, Germany).

The cultivation medium is mixed by capillary waves induced by the vertical oscillation of the cwMBR, as described by Frey et al. [20,44] and Meinen et al. [21]. Therefore, the cwMBR mounting was placed on a quadratic polyvinyl chloride platform with an electromagnetic exciter (Ex 45 S, Visaton, Haan, Germany) under each corner. The audio signal, which triggers the exciters, is generated by oscilloscope software (Soundcard Scope, Zeitnitz, Germany). Afterwards, the signal is transmitted by a soundcard (Gigaport HD+, ESI Audiotechnik, Leonberg, Germany) and enhanced by amplifiers (M034 N, Kemo Electronic, Geestland, Germany) to generate vertical oscillation.

To ensure stable cultivation conditions, the cwMBR platform was placed in an incubation chamber, which was tempered by a thermostat (Eco E4, Lauda Dr. R. Wobser, Lauda-Königshofen, Germany) connected to a Pt100 element and a heat exchanger (Hydro Series H55, Corsair Components, Fremont, CA, USA). A constant humidity of 95% in the incubation chamber was maintained by an ultrasonic humidifier (DH-24B, Conrad Electronic, Hirschau, Germany).

### 2.2. Integration of Optical Sensors

Optical sensors for pH, glucose and DO measurements were spotted on a transparent 13 µm thick PET foil, which was glued on the cwMBR bottom with light-activatable and biocompatible glue (Loctite AA 3301 LC, Henkel, Düsseldorf, Germany). Light absorbance was measured by a miniaturized spectrometer (Flame UV-Vis Spectrometer, Ocean Optics, Orlando, Florida, USA) and a blue LED (λ_max_ ≈ 485 nm) as the light source.

The optical pH sensor consisted of (Z)-4-(5-((5-(4-hydroxyphenyl)-3-phenyl-2H-pyrrol-2-ylidene)amino)-4-phenyl-1Hpyrrol-2-yl)benzoic acid and Egyptian Blue as a reference dye. Oxygen-sensitive particles for the oxygen and glucose sensor consisted of poly-tert-butylstyrene stained with 2% (*w*/*w*) platinum (II) meso-tetra(4-fluorophenyl) tetrabenzoporphyrin as a sensor dye. All sensor dyes were synthesized as described previously [32,37,45]. The sensor matrix consisted of an ether-based hydrophilic urethane hydrogel (HydroMed D4, AdvanSource Biomaterials, Wilmington, MA, USA).

All sensor spots were manufactured from sensor cocktails containing all ingredients, which were spotted on the PET foil. After evaporation of the solvent, a sensor spot with an average diameter of 0.5 mm remained. For sensor spotting, a piezoelectric microdispenser (MDV 3200A, Vermes Microdispensing, Holzkirchen, Germany) was used as a spotting tool. For precise spotting, the microdispenser was connected to a CNC microstep driver (Triple Beast, Benezan Electronics, Rottenburg, Germany) and a step motor for single-axis movement (Axis Motor, Isert-Electronic, Eiterfeld, Germany).

For the pH sensor, 0.1 mg of the sensor dye, 16.8 mg of Egyptian Blue and 100 mg of Tetrahydrofuran were diluted in 415 mg of hydrogel solution (8% (*w*/*w*) in water/ethanol (1:10) as a sensor cocktail. The solution was homogenized ultrasonically.

The oxygen sensor cocktail was prepared by dissolving 37 mg of oxygen-sensitive particles in 413 mg of water/ethanol (1:10). Furthermore, 330 mg of hydrogel solution was added (21% (*w*/*w*) to water/ethanol (1:10).

The glucose sensor consists of a sensor layer made of oxygen-sensitive particles and cross-linked glucose oxidase (GOx) aggregates in polyurethane hydrogel covered by a diffusion barrier made of polyhydroxyethylmethacrylate (pHEMA; Polysciences, Niles, IL, USA). For increased enzyme stability, GOx (glucose oxidase from Aspergillus niger, Merck, Darmstadt, Germany) was cross-linked using glutaraldehyde (Merck, Darmstadt, Germany). Therefore, 2.2 mg of GOx was diluted in 41 µL of deionized water and precipitated by adding 140 µL of ethanol. For enzyme cross-linking, 5.5 µL of glutaraldehyde was added to the enzyme precipitates, and the solution was stored at room temperature under constant movement overnight. Afterwards, a mixture of 38 mg of polyurethane hydrogel and 25 mg of oxygen-sensitive particles in 450 µL ethanol and 15 µL of deionized water was added. The diffusion barrier cocktail consisted of 75 mg of pHEMA in 540 µL of deionized water and 171 µL of ethanol. The microdispenser settings can be found in Appendix B.

### 2.3. Sensor Read-Out and Data Analysis

Sensor read-outs were obtained using a fiber-optics read-out device (FireStingO_2_ (oxygen and glucose sensors) and FireSting Pro (pH sensor), Pyroscience, Aachen, Germany). FireStings and the spectrometer for absorbance measurement were connected to the cwMBR by optical fibers (FT400UMT, Thorlabs, Newton, New Jersey, USA) and ST connectors (Telegärtner, Steinenbronn, Germany). A conventional 5 mm blue LED was placed in the lid above the cwMBR as the light source for transmission measurement (Figure 2c).

The calibration of all sensors was performed at cultivation conditions of atmospheric pressure, a cultivation temperature of 37 °C, cultivation medium in the cwMBR and switched-on oscillation of the cwMBR. Oxygen and glucose sensors were calibrated for different oxygen saturations, as both sensors measure oxygen saturations within the sensor. Therefore, two-point calibration with air-saturated and oxygen-free medium was performed. Anoxic conditions were achieved by gassing the cwMBR headspace with humidified nitrogen. Air saturation was achieved by gassing the headspace with humidified air.

After oxygen calibration, the glucose sensor was calibrated using seven different glucose concentrations between 0 and 30 mM. The resulting glucose concentration can be calculated with the help of a linear fit in the ∆*p_O2_*-*c_glucose_* diagram.

The pH sensor was calibrated by the stepwise addition of 35 nL of hydrochloric acid (1 M) to the cwMBR containing M9 medium with an initial pH value of 8.07. Hydrochloric acid was added to the cwMBR using a microdispenser (PipeJet Nanodispenser, BioFluidix, Freiburg, Germany). The addition of hydrochloric acid resulted in pH values between 8.07 and 6.07, which was proven in a previous experiment by adding equivalent ratios of acid to 100 mL of cultivation medium and measuring the pH value with a pH electrode (CG840, Schott, Mainz, Germany).

Absorbance measurement was used to monitor the cell growth of *E. coli*. This is a widespread method to quantify biomass growth in MBRs [16,46,47,48,49,50]. Therefore, a blue LED was placed in the cwMBR lid, and the transmission light intensity was measured by a spectrometer via optical fibers (Figure 2c). The absorbance was calculated by the Beer–Lambert law using the light intensity at the beginning of the cultivation *I*_0_ and the actual light intensity *I*_1_:

(1) $$E_{\lambda }=log_{10}\frac{I_{0}}{I_{1}}$$

Usually, *I*_0_ represents the transmitted light intensity without biomass. This would require opening the cwMBR mounting lid and measuring the volume change of the cwMBR droplet, resulting in slight position changes of the lid and the integrated LED. As a consequence, the transmitted light intensity would change. Therefore, *I*_0_ represents the transmitted light intensity at the beginning of the cultivation. For a better comparison between the cwMBRs, the absorbance was normalized to the maximal value.

### 2.4. Sensor Characterization

Since the oxygen and the pH sensors have previously been characterized in different applications [8,11,20,32,37,41,44,45], the sensor characterization focuses on the glucose sensor, which has not been previously described.

The dynamic range is one of the most important parameters for sensor characterization, as it defines possible applications. The dynamic range was investigated by filling the cwMBR with 7 µL of medium with glucose concentrations between 0 and 30 mM and measuring ∆*p_O2_*, which describes the oxygen partial pressure difference between oxygen and glucose sensors. The linear range in the ∆*p_O2_*-*c_glucose_* diagram is the dynamic range of the sensor. As GOx in the sensor is inactivated continuously during application, the sensor stability was analyzed for the stability of the sensor signal. Therefore, the cwMBR was filled with 10 mM glucose solution (in 100 mM phosphate buffer), and ∆*p_O2_* was measured over three days. The glucose solution was replaced twice a day. Therefore, the effect of evaporation on the glucose concentration can be neglected.

The response time of the sensor (*t*_90_) was determined by measuring the time that the sensor needed to obtain a constant signal after adding a 10 mM glucose solution.

As GOx in the glucose sensor oxidizes glucose using oxygen, the availability of oxygen is essential for the functionality of the sensor. To investigate the influence of limited oxygen availability, the cwMBR was filled with different glucose solutions between 0 and 30 mM and gassed with different air–nitrogen ratios, and ∆*p_O2_* was measured. The nitrogen ratio varied between 0 and 75%. Furthermore, the influence of pH changes on the glucose sensor was measured by the addition of glucose-containing phosphate buffer with pH values between 6 and 8 in the cwMBR.

Due to the oxidation of glucose to gluconolactone by GOx, the glucose sensor consumes glucose. This glucose consumption was quantified by incubating the sensor in the cwMBR with 7 µL of 20 mM glucose solution in phosphate buffer for 24 h. The remaining glucose concentration was determined by high-performance liquid chromatography (HPLC) afterwards (see Appendix A).

In this work, the previously applied scattered light measurement was replaced by absorbance measurement, as absorbance measurement provides results with less background noise and lower standard deviation. Hence, the results were more stable. Therefore, the absorbance sensor system was first characterized regarding its applicability. For this characterization, different droplets of *E. coli* suspension in LB medium with optical densities (ODs) between 0.5 and 3.5 were placed in the cwMBR, and the absorbance was measured. The normalized absorbance was calculated using Equation (1) (see Section 2.3) and normalized to 100% afterwards.

### 2.5. Cultivation of Escherichia coli

As proof of concept for the application of the optical sensors in biotechnological cultivations, the cwMBR with integrated sensors was applied to monitor the growth and substrate consumption of *E. coli* BL21 (DE3) pMGBm41. A shake flask culture of *E. coli* was inoculated by adding 10 µL of cryopreserved cells to 25 mL of complex medium containing 10 g/L soy peptone, 5 g/L yeast extract and 5 g/L sodium chloride (all purchased from Carl Roth, Karlsruhe, Germany) in a 250 mL shake flask with three baffles. The culture was incubated at 37 °C and 200 min^−1^ (shaking diameter 5 cm) overnight (Certomat IS, Sartorius, Göttingen, Germany).

The cwMBR culture was inoculated from the shake flask culture, which was diluted to an OD of 0.1 with M9 minimal medium (Table A1; Appendix A). Cultivation in the cwMBR was performed according to Frey et al. [20]. Therefore, the diluted *E. coli* culture was transferred to the cwMBR and incubated under vertical oscillation of the cwMBR. The cwMBR oscillated with a frequency of 70 Hz and an intensity of 5% at 37 °C and a relative humidity of 95%. During cultivation, biomass concentration, pH value, oxygen saturation and glucose concentration were measured by optical sensors.

For a comparison to cwMBR cultivation, *E. coli* was also cultivated in shake flasks. The procedure for this cultivation can be found in Appendix A.

## 3. Results and Discussion

### 3.1. Sensor Characterization

Sensors are essential to characterize cultivation in the cwMBR, as the small volume of 7 µL limits the ability to take samples. Therefore, four parameters were defined as essential for process characterization: biomass, glucose, oxygen and pH. In contrast to biomass and glucose sensors, oxygen and pH sensors have previously been described, characterized and applied in different systems [8,11,20,32,37,41,44,45]. Hence, this section focuses on the characterization of the biomass and glucose sensors.

#### 3.1.1. Biomass Sensor

**Figure 3:**
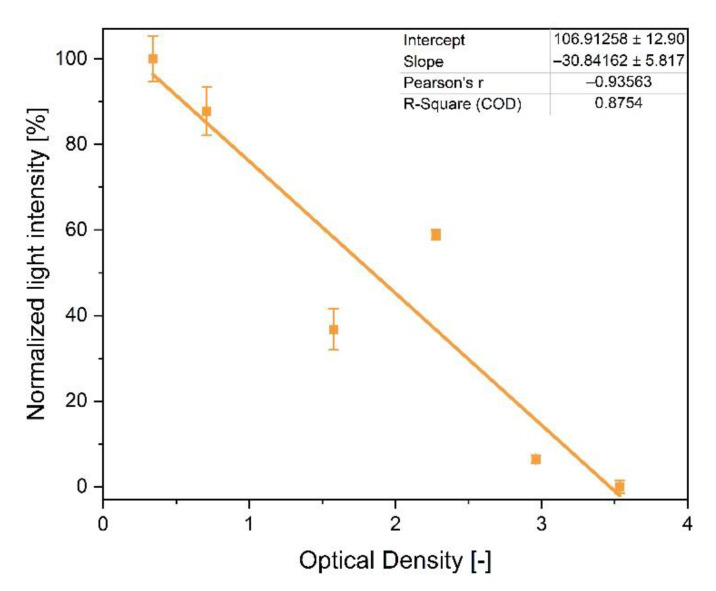
Normalized light intensity, measured by a spectrometer, of *E. coli* culture suspensions with optical densities between 0.5 and 3.5 in the cwMBR. A blue LED over the cwMBR was used as the light source, and a spectrometer was used as the read-out device.

The measurement of biomass by quantifying transmission light intensity is a widespread method to monitor cell growth in MBRs [16,46,47,48,49,50]. Compared to scattered light measurement, as performed by Frey et al. [20], absorbance measurement has been revealed as a more stable method for biomass measurement with less background noise and lower standard deviation. Therefore, a blue LED as the light source was placed over the cwMBR, and transmission light intensity was measured using a USB spectrometer connected to the cwMBR bottom by optical fibers (Figure 2c). For the characterization of the biomass sensor, the normalized light intensity of the cwMBR filled with *E. coli* culture suspensions with varying ODs was measured. The data were plotted against the OD measured by a standard photometer (Libra S11, Biochrom Ltd., Cambridge, UK), indicating linearity between the two datasets (Figure 3). Hence, OD measurement can be applied for the quantification of biomass in the cwMBR. This measurement system was applied as an online tool for growth measurement in further cultivations and was revealed to be more stable compared to the previously applied scattered light measurement.

#### 3.1.2. Glucose Sensor

**Figure 4:**
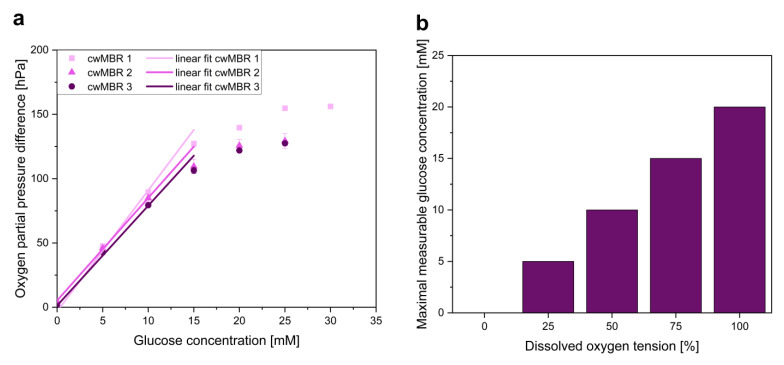
(**a**) Oxygen partial pressure difference (∆*p_O2_*) between oxygen and glucose sensors in the cwMBR with linear fit. Glucose solutions between 0 and 30 mM in M9 minimal medium were used. The results indicate proportionality between ∆*p_O2_* and the glucose concentration within the dynamic range; (**b**) maximal measurable glucose concentration of the sensor at dissolved oxygen tensions between 0 and 100%. The results illustrate an increasing dynamic range with rising oxygen availability.

Glucose is often applied as a carbon source in biotechnological cultivations. Therefore, its quantification in microscale cultivations is also of special interest. The measurement principle of the glucose sensor is based on the oxygen demand of GOx during the oxidation of glucose. Therefore, a sensor with phosphorescent oxygen-sensitive particles was modified by the addition of cross-linked GOx and a diffusion barrier for glucose. To measure the glucose concentration, the oxygen partial pressure difference between the glucose sensor and a separated optical oxygen sensor (∆*p_O2_*) was measured (Figure 1). This sensor signal is proportional to the glucose concentration in the surrounding cultivation medium (Figure 4a). The linearity of the ∆*p_O2_*–concentration graph defines the linear range of the sensor. In this example, the glucose sensors can measure up to 15 mM. Glucose concentrations above 15 mM do not further increase ∆*p_O2_*, and therefore, the maximal measurable glucose concentration is reached. Nevertheless, modification of the diffusion barrier and enzyme concentration can increase the dynamic range of the sensor. Therefore, variations in the dynamic range of the glucose sensor were observed due to statistical errors in the number of enzyme aggregates in the sensors.

Besides the dynamic range, other parameters, including stability, pH value, oxygen availability, response time and glucose consumption, of the glucose sensor had to be analyzed to review its applicability.

First, the sensor stability was investigated. The glucose sensor contains the enzyme GOx, which is inactivated during cultivation due to hydrolysis, which decreases the stability of the whole sensor [51]. Besides hydrolysis, GOx produces hydrogen peroxide, which further limits the activity of GOx [52]. The inactivation of GOx leads to declining partial pressure differences between the oxygen and glucose sensors during cultivation. This decrease was quantified at 13.75 ± 7.08 hPa during an 8 h cultivation time. However, the measurement of GOx activity during cultivation revealed a linear decrease in enzyme activity. Therefore, the inactivation can be corrected mathematically with a second calibration of the glucose sensor after application. Furthermore, the long-term stability of the sensor was investigated. Therefore, the cwMBR was incubated with a 10 mM glucose solution for 67 h, and ∆*p_O2_* was measured twice a day after refilling the cwMBR with a new 10 mM glucose solution. A linear decrease from 108 to 36 hPa was observed, indicating a decreasing sensor signal. However, this decrease can be corrected mathematically.

As GOx was inactivated during the application of the glucose sensor, a significantly decreased ∆*p_O2_* value was measured at the beginning of the second application compared to the first application. It further decreased during the second application. This led to decreased sensor accuracy. Therefore, a single-use application of the glucose sensor is recommended.

As enzymatic reactions are influenced by environmental factors, e.g., temperature, pH value and substrate concentration [32,51,53,54,55], it is expected that these factors also influence ∆*p_O2_*. However, the temperature influence on the glucose sensor can be neglected in bioprocesses, as the temperature is kept constant in bioreactors, including the here applied cwMBR.

**Figure 5:**
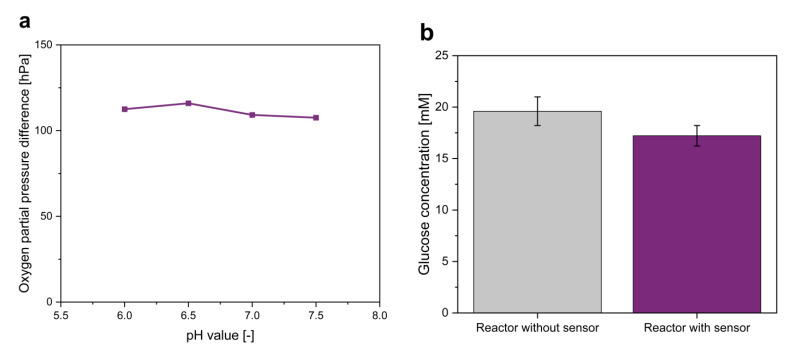
(**a**) Oxygen partial pressure difference between oxygen and glucose sensors of a 10 mM glucose solution in phosphate buffer at different pH values. The results indicate neglectable pH influence at relevant pH values; (**b**) glucose concentration determined via HPLC in cwMBRs after incubation with or without a glucose sensor after 24 h with an initial glucose concentration of 20 mM. The results illustrate the low glucose consumption of the optical glucose sensor.

In contrast to the temperature, the pH value varies during the cultivation of *E. coli* in the cwMBR due to the production of acetic by-products such as acetate [35]. Therefore, investigation of the influence of pH changes on the glucose sensor is important before application. The highest sensor activity is expected at pH 6.5, as it is the pH optimum of the here applied GOx [55]. Further investigations indicated pH decreases of 0.5 pH units during the cultivation of *E. coli* in the cwMBR (see Section 3.2.3). However, these pH shifts only increased the sensor signal by 1.5% and therefore can be neglected (Figure 5a). As a consequence, no pH dependency was considered during applications with low pH changes.

As GOx needs oxygen as a substrate for glucose oxidation, its availability strongly influences the performance of the sensor. Decreasing DO tensions reduced the dynamic range of the sensor and therefore the maximal measurable glucose concentration (Figure 4b). Bisection of the available DO decreased the dynamic range by 50%. Due to the high oxygen input in the cwMBR with *k_L_a* values of up to 340 h^−1^, the maximal measurable glucose concentration is not reduced, even in high-oxygen-demanding bioprocesses such as *E. coli* cultivations (see Section 3.2.2), and therefore does not limit the applicability of the optical glucose sensor in the cwMBR.

Another important parameter of the glucose sensor characterization is the response time, as the sensor has to be fast enough to measure concentration changes. Therefore, the *t*_90_ value of the sensor was determined, which describes the time that is needed to reach 90% of the final sensor signal. The *t*_90_ value for concentration changes of 10 mM was determined to be 6.4 min ± 2.0 min. As a comparison, the glucose demand of fast-growing *E. coli* was determined under optimal growth conditions in a shake flask (see Section 3.2.3). In this cultivation, *E. coli* needed 85 min for similar concentration changes, indicating sufficient response time of the sensor even for fast-growing microorganisms such as *E. coli*.

The GOx in the glucose sensor catalyzes the oxidation of glucose to gluconolactone. As a consequence, the glucose concentration in the cultivation medium declines through the sensor. In large-scale bioreactors, it is expected that this effect can be neglected due to the high amount of glucose in the cultivation medium. However, the glucose sensor is applied in a cwMBR with a volume of only 7 µL, giving the glucose consumption of the sensor a higher relevance. Therefore, the glucose consumption of the sensor of a 20 mM glucose solution during a 24 h period was determined via HPLC (Figure 5b). Compared to a cwMBR without a glucose sensor, the glucose concentration in the sensor-equipped cwMBR declined by 2.4 mM, resulting in glucose consumption rates of 0.1 mmol ∙ L^−1^ ∙ h^−1^. These concentration changes have to be considered during further interpretation of the sensor results, even though the performed *E. coli* cultivations last for only one-third of the here described experimental time. Furthermore, the glucose concentration during cultivation is lower and even declines. This results in even lower glucose consumption rates, as GOx shows typical Michaelis–Menten behavior with decreasing turnover rates at declining substrate concentrations without substrate inhibition [56].

### 3.2. Application of Optical Sensors in cwMBR Cultivation

After characterization, the optical sensors were applied as tools for bioprocess monitoring of an *E. coli* cultivation in the cwMBR. The determination of process parameters such as biomass concentration, pH value, oxygen saturation or glucose concentration allows the characterization of the process, which is fundamental for the application of MBRs for screening, process development or cell-based assays. Optical sensors are a promising tool, as they provide non-invasive measurement, have a small size and are cost-effective. Due to the low volume, sampling is often not possible in MBRs.

#### 3.2.1. Biomass Measurement via Transmission Light Intensity

To monitor cell growth in the cwMBR, a biomass sensor based on light absorbance measurement was integrated in the cwMBR. Three parallel cultivations including biomass measurement were performed to prove the suitability of this sensor system for the cwMBR. Absorbance measurement was performed using a blue LED as the light source and a miniaturized spectrometer as the read-out device.

**Figure 6:**
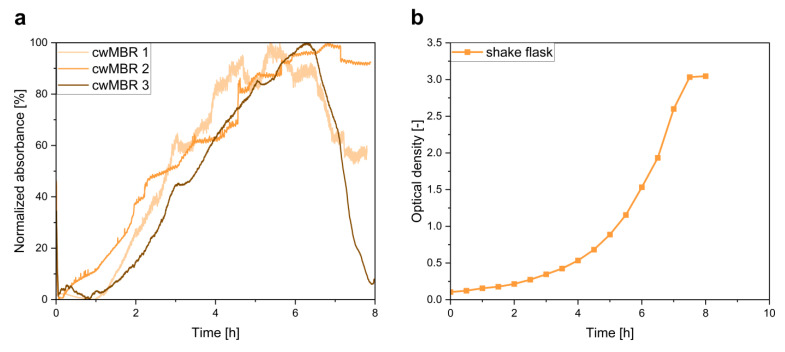
(**a**) Normalized absorbance of an *E. coli* cultivation in M9 minimal medium in three parallel cwMBRs showing cell growth in all cwMBRs. Vertical oscillation of the cwMBR platform was performed at 70 Hz and an amplitude of 5%. The absorbance was measured using a blue LED and a miniaturized spectrometer; (**b**) optical density of *E. coli* cultivated in shake flasks with M9 medium. The graph shows the mean of a triplicate.

The normalized absorbance in three parallel cwMBRs during the cultivation of *E. coli* in M9 minimal medium is presented in Figure 6a. As a comparison, *E. coli* was also cultivated in shake flasks under similar growth conditions.

After a short lag-phase of approximately 1 h in two cwMBRs, the absorbance and therefore the biomass concentration in all three cwMBRs increased significantly for the next 5 to 6 h. Afterwards, no further increase in absorbance was observed, and it started to decline in two cwMBRs. The decrease at the end of the cultivation can be explained by slight position changes in either the lid, the integrated LED or the optical fibers, leading to light intensity changes. In general, a similar increase in absorbance was detected in all three parallel cwMBR, which proves the suitability of this method for biomass monitoring in the cwMBR. Furthermore, similar cell growth behavior to that reported by Frey et al. [20] was observed. In contrast to the absorbance measurement, Frey et al. [20] applied a scattered light measurement for biomass determination, which was less stable than the absorbance measurement in the cwMBR. Compared to the OD measurement in the shake flask (Figure 6b), different growth behavior was observed. While the biomass grew linearly in the cwMBR, typical exponential growth behavior was observed in the shake flask. The linear growth can be explained by growth limitations due to small concentration changes resulting from slight evaporation of the cultivation medium. Hence, the evaporation leads to increased concentrations of growth-limiting substances such as acetate. Nevertheless, a similar duration of cell growth was observed in both systems, indicating the suitability of the sensor system for absorbance measurement and therefore offering a reliable tool for biomass determination.

Besides the quantification of cell growth, the setup can be applied for future colorimetric and fluorescence-based assays in the cwMBR with only slight modifications. By applying specific light sources with filters, absorption at a specific wavelength or fluorescence intensity measurement can be performed in the cwMBR, enabling, e.g., viability assays such as the XTT assay [57]. This allows the application of the cwMBR for a broad range of applications, including toxicity studies in biopharmaceutical research.

#### 3.2.2. Optical Glucose and Dissolved Oxygen Measurement

Glucose and dissolved oxygen concentrations are also important process parameters to be analyzed in cwMBR cultivations. Both analytes are measured by optical sensors containing oxygen-sensitive particles in a hydrogel. By measuring the oxygen partial pressure difference between the oxygen and glucose sensors, the glucose concentration can be calculated (Figure 1).

**Figure 7:**
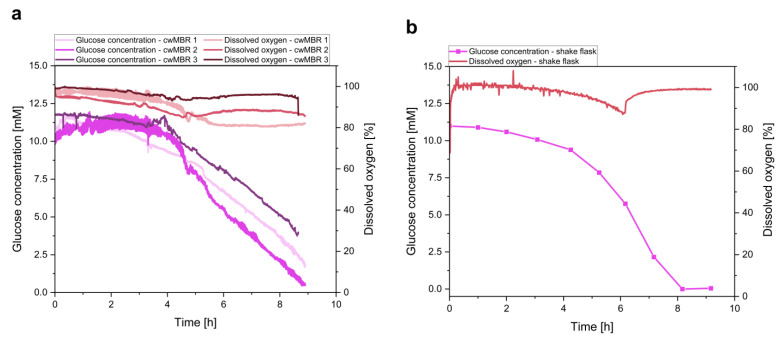
(**a**) Glucose concentration and dissolved oxygen tension of an *E. coli* cultivation in M9 minimal medium in three parallel cwMBRs, showing consumption of both analytes in all cwMBRs. Vertical oscillation of the cwMBR was performed at 70 Hz and an amplitude of 5%. Both analytes were measured by optical sensors on the bottom of the cwMBR; (**b**) glucose concentration and dissolved oxygen tension of a cultivation of *E. coli* in shake flasks with M9 medium. The graph shows the mean of triplicates.

Equivalent to the biomass measurement, glucose was measured in three parallel cwMBR cultivations of *E. coli* in M9 medium. The read-out of the sensors was performed by a fiber-optics read-out device connected to the cwMBR by optical fibers. Before and after cultivation, the cwMBR was calibrated with M9 medium containing glucose concentrations between 0 and 15 mM (Figure 3). The calibration after application increases the accuracy, as it compensates for GOx inactivation during cultivation (see Section 3.1.2). As a comparison, *E. coli* was also cultivated in shake flasks, and measurements, including glucose measurement by HPLC and oxygen measurement by optical sensors, were performed (Figure 7b).

The glucose concentration and dissolved oxygen tension of the cultivation are presented in Figure 7a. It is clearly visible that the glucose consumption of *E. coli* during cultivation was measured by the optical sensors. After a lag phase of approximately 4 h, the glucose concentration started to decline linearly, which is the expected inverse behavior to the biomass concentration discussed previously. Compared to the shake flask cultivation, a similar glucose consumption can be observed. After 4 h, a significant decrease in the glucose concentration was measured. However, the glucose concentration in the cwMBR did not decline exponentially, which could be observed in the shake flask. This linear decrease in the cwMBR correlates with the linear biomass growth in the cwMBR (Figure 6a) and with results from *E. coli* cultivations in the cwMBR described by Frey et al. [20]. This can be explained by growth limitations. Hence, the glucose is not fully consumed at the end of the cultivation after approximately 9 h. Nevertheless, these results indicate the applicability of the here described optical glucose sensor for cwMBR cultivations. Furthermore, this proof of concept of a novel optical glucose sensor suggests that it can be applied for more complex cultivations in different types of microscale cultivation systems.

In addition to the glucose concentration, the oxygen saturation was measured in both systems, indicating a high oxygen saturation above 80% in cwMBRs (Figure 7a) as well as in shake flasks (Figure 7b) during the whole cultivation period. The high oxygen saturation can be explained by the sufficient oxygen input in both systems. However, at more favorable growth conditions in complex media or in fed-batch cultivations with higher oxygen demand, the cwMBR is expected to provide better growth conditions due to the higher *k_L_a* value of 340 h^−1^ [20] compared to shake flasks with *k_L_a* values far below 100 h^−1^ [58].

#### 3.2.3. Optical pH Sensor

As most microorganisms produce acidic or alkalic by-products such as acetate, which can have a negative influence on cell growth [35], pH measurement is important to characterize cultivations in bioreactors. For pH measurement, an optical pH sensor based on aza-BODIPY dyes was integrated in the cwMBR. Deprotonation of the sensor dye leads to fluorescence quenching, which is measured. The sensor was characterized previously [33]. In this paper, the sensor was applied for the characterization of an *E. coli* cultivation in the cwMBR with M9 medium as proof of concept for pH measurement in the cwMBR. As a comparison, *E. coli* was also cultivated in a shake flask, and the pH value was measured by a pH electrode.

**Figure 8:**
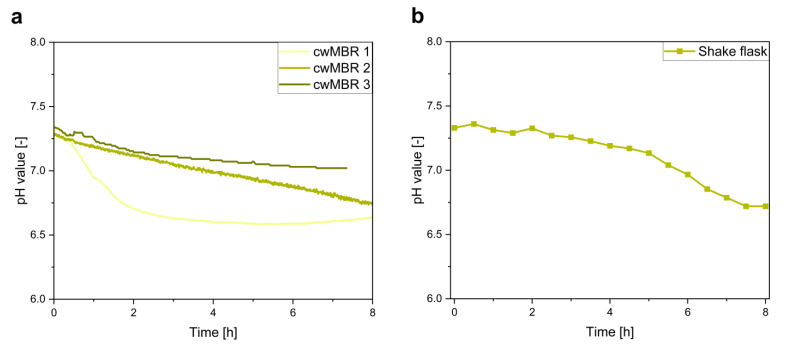
(**a**) pH values of an *E. coli* cultivation in M9 minimal medium in three parallel cwMBRs, showing a pH decrease due to the production of acidic by-products in all cwMBRs. Vertical oscillation of the cwMBR was performed at 70 Hz and an amplitude of 5%. pH was measured by optical sensors on the bottom of the cwMBR; (**b**) pH value of a cultivation of *E. coli* in shake flasks with M9 medium measured by a pH electrode. The graph shows the mean of triplicates.

As expected, acidic by-product formation during the cultivation led to a pH decline in the cwMBR (Figure 8a). It decreased from an initial pH value of 7.3 to a final pH of 6.6 to 7. The differences between the cwMBRs can be explained by slightly different growth conditions resulting from the slight evaporation of the cwMBR droplet, which was previously observed during the investigation of the absorbance (Figure 6a) and glucose concentration (Figure 7a). Nevertheless, a similar pH profile to that of the shake flask cultivation (Figure 8b) can be observed. The pH value in the shake flask declined during the whole cultivation period due to the production of acidic by-products to a final pH value of 6.7, which is comparable to the cwMBR cultivations. These results indicate the excellent applicability of the optical pH sensor for pH measurement in the cwMBR. 

## 4. Conclusions

This work describes the integration of optical sensors for biomass, glucose, dissolved oxygen and pH in a cwMBR with a volume of only 7 µL. The glucose sensor uses novel technology for bioprocess characterization based on oxygen consumption at the microscale, which has only been applied for blood glucose measurement or large-scale glucose measurement so far. Amongst others, the glucose sensor was characterized in terms of the dynamic range, stability and response time prior to application. Furthermore, the cwMBR was equipped with an absorbance sensor, which provides more stable results than the previously applied scattered light sensor with less background noise and lower standard deviation. All sensors were successfully applied for the characterization of an *E. coli* cultivation as proof of concept for bioprocess monitoring in the cwMBR. Therefore, biomass, dissolved oxygen, glucose and pH were measured in the cwMBR and compared to an *E. coli* shake flask cultivation.

To the best of our knowledge, there is no MBR described in the literature that combines a volume in the lower microliter scale with sensors for the measurement of four important process parameters. Hence, the advantages of small-scale MBRs, including the saving of medium and test substances, simple automatization and high parallelizability, are extended by a high degree of process information. Therefore, the cwMBR with integrated optical sensors is a promising alternative to larger MBRs for applications with cost-intensive media components or less available testing substances, as it helps to reduce the needed amount of these substances without losing process information. By cultivating mammalian cell cultures, the cwMBR might also be applied for cell-based assays, such as toxicity studies, which can also be characterized by the integrated sensor. Overall, the cwMBR is a promising tool for different applications in the field of biopharmaceutical and bioprocess research.

## Data Availability

The data presented in this study are available on request from the corresponding author.

## Appendix A. Shake Flask Cultivation of *E. coli*

As a comparison to cwMBR growth, *E. coli* was cultivated in a shake flask culture. Therefore, a pre-culture from cryo-preserved cells was grown in 50 mL of LB medium in 500 mL shake flasks with four baffles. The pre-culture was incubated overnight at 37 °C at a shaking frequency of 200 min^−1^ (shaking diameter: 5 cm) in an ISF1-X incubator (Kühner, Birsfelden, Switzerland). The main culture was performed in triplicate under identical conditions in M9 minimal medium (Table A1).

The cultivation was sampled hourly and analyzed for OD, pH and glucose concentration. Furthermore, the DO was measured online by optical sensors in a separated shake flask (SFR Shake Flask Reader, Presens Precision Sensing, Regensburg, Germany). The OD was determined by a photometer (Libra S11, Biochrom, Camebridge, UK), and the pH value was measured by a pH electrode (CG840, Schott, Mainz, Germany). Glucose was quantified by HPLC (Hitachi LaChrom Elite, Tokio, Japan), which was equipped with a Metacarb 87C column (Varian, Palo Alto, CA, USA). Ultrapure water was used as the mobile phase with a flow rate of 0.6 mL/min and a column temperature of 85 °C. Glucose was measured by a refractive index detector at a retention time of approximately 10 min.

**Table A1:** Components and their concentrations in M9 minimal medium (all purchased from Carl Roth, Karlsruhe, Germany).

Component	Concentration [mg/L]
Na_2_HPO_4_ ∙ 2 H_2_O	7520
KH_2_PO_4_	3000
NaCl	500
NH_4_Cl	500
Glucose	3600
MgSO_4_	120
CaCl_2_	33
Biotin	1
Thiamin	1
EDTA	50
FeCl_3_ ∙ 6 H_2_O	0.0083
ZnCl_2_	0.84
CuCl_2_ ∙ 2 H_2_O	0.13
CoCl_2_ ∙ 2 H_2_O	0.1
H_3_BO_3_	0.1
MnCl_2_ ∙ 4 H_2_O	1.6

## Appendix B. Sensor Spotting

For process characterization, optical sensors for pH, oxygen and glucose were spotted on the bottom of the cwMBR (Figure 2b), as described in Section 2.2. The settings for the microdispenser are displayed in Table A2.

**Table A2:** Microdispenser settings for the preparation of the dissolved oxygen, pH and glucose sensor.

	Dissolved Oxygen Sensor	pH Sensor	Glucose Sensor (Sensor Cocktail)	Glucose Sensor (Diffusion Barrier)
Tappet lift	35%	80%	30%	65%
Rising time	0.5 ms	0.3 ms	0.2 ms	0.2 ms
Open time	0.1 ms	0.1 ms	0.1 ms	0.2 ms
Falling time	0.06 ms	0.08 ms	0.12 ms	0.07 ms
Delay	0.1 ms	0.1 ms	0.1 ms	0.1 ms
Number of pulses	1	4	3	3
Pressure	1000 mbar	400 mbar	200 mbar	300 mbar
